# Cross-Border E-Commerce Intelligent Information Recommendation System Based on Deep Learning

**DOI:** 10.1155/2022/6602471

**Published:** 2022-02-23

**Authors:** Liuqing Li

**Affiliations:** 1 Huanghuai University, Zhumadian, 463000, China

## Abstract

In order to improve the effect of cross-border e-commerce intelligent information recommendation, this paper applies deep learning to the intelligent information processing and intelligent recommendation of e-commerce and proposes an improved version of the topic model to solve the problem of feature extraction of the text of the recommendation system. In order to deal with translation problems, this paper proposes an end-to-end sequence-to-sequence learning method. In addition, this study uses the long tail theory to excavate the mass commodities in the niche and recommends these products to users as suggestions. Finally, this paper proposes a niche product recommendation algorithm based on the graph search strategy based on the graph model. The experiment shows that the cross-border e-commerce intelligent information recommendation system based on deep learning proposed in this paper has a good recommendation effect and meets the recommendation needs of cross-border e-commerce.

## 1. Introduction

This has stimulated offline entities to a certain extent and has formed an integrated online and offline sales strategy. With the increasing scale of online business platforms, recommendation systems have also risen [1] and have become one of the backbone technologies in various fields today. It is also mainly from the perspective of “people-oriented” to make the originally large and complex assembly line more intelligent, which greatly facilitates human beings. Therefore, it is favored by many scholars at home and abroad, and more and more researchers invest in this field [2].

Nowadays, traditional foreign trade is in a state of insufficient growth and continued sluggishness. However, cross-border e-commerce has sprung up and has experienced a “blowout” growth, which has become an important driving force for industrial upgrading and new growth in foreign trade under the new normal of the economy [3].

Banks and other financial institutions jointly established a series of norms and mechanisms based on the collection, processing, credit rating, credit supervision, and rewards and punishments related to credit information, thereby effectively enhancing the trust relationship and creditworthiness between transaction entities. Optimize the credit environment of cross-border e-commerce The system's data analysis center converts it into indicators in some way, obtains the credit rating of the transaction subject through system analysis, and provides a basis for system supervision, rewards, and punishments.

This study applies deep learning to the cross-border e-commerce intelligent information recommendation system to overcome the geographical problems of cross-border e-commerce and improve the recommendation effect of cross-border e-commerce.

## 2. Related Work

The rule-based recommendation algorithm is mainly based on customer-defined rules for information association and information mining and then recommends and analyzes customers through existing rules. The advantage of this algorithm is simple and fast [4].

The recommendation based on content filtering is to make recommendations by comparing the customer profile with existing resources. The key consideration of this recommended technique is the calculation of similarity. Because most customers are unable to accurately analyze and accurately express their needs, therefore, the rule-based recommendation algorithm has big flaws. The content-based recommendation technology compares the customer's feature description file with the resource file and searches for similar word features. Typical systems include PersonalWebWatcher [5], WebMate [6], and WebACE [7].

The recommendation algorithms of collaborative filtering technology include user-based collaborative filtering. Among them, user-based collaborative filtering is realized by analyzing the similarity of user characteristics and behaviors. The item-based collaborative filtering method analyzes the similarity relationship between items and then provides users with similar items. The model-based collaborative filtering is based on recommendations which are based on the relationship between existing item features and user features [8].

Hybrid filtration technology: since the content based have their own advantages and disadvantages, in many practical applications, these two different recommendation algorithms are combined for recommendation, which is a hybrid recommendation algorithm [9]. Huang et al. [10] use the Bayesian probability framework to combine content-based features and collaborative filtering features for recommendation calculations and achieve better results. Liang and Qin [11] combine the two recommendation techniques in a weighted form, thereby increasing the diversity of recommendation results. Liu et al. [12] combined model-based collaborative filtering and memory-based model for recommendation and then recommended products effectively. Chen [13] applies the ranking learning algorithm to the hybrid recommendation algorithm for model fusion. Generally speaking, such an algorithm structure combines the advantages of content-based recommendation algorithm, but increases the complexity of the algorithm and the running time cost. Recommendation algorithms play a huge role in all areas of human life and work. However, although there are various differences in its algorithms, it must be admitted that each algorithm has its own advantages and applicable characteristics. There is still no algorithm that is close to perfect because various algorithms have their unavoidable defects to a certain extent. Therefore, how to effectively analyze and integrate these algorithm resources so as to combine their strengths and avoid weaknesses as much as possible and then combine their advantages for recommendation has become one of the optimization directions of recommendation algorithms.

He [14] proposed a novel interpretation interface, which focuses on the integration of extracting characteristic emotions and static attributes from product reviews to help users explore and understand the product space more effectively and then learn more from other customers' experiences and explain the product preferences of target users well. Xu et al. [15] proposed a multitask learning interpretable recommendation algorithm. The algorithm uses tensor decomposition to integrate the user preference modeling in the recommendation task and the modeling of the opinion content in the interpretation task. It can not only predict the target user's preferences, for items can also provide explanations about the characteristics of specific items. Wei et al. [16] propose a recommendation method that uses tags as features and explain to the user why the recommended movie is related to him based on the features. At the same time, user research experiments have been conducted, and the results show that providing feature-based explanations for the recommendation results can help improve the effectiveness of the recommendation results. Hosseini et al. [17] also verified through user research experiments that feature-based explanations are closely related to user satisfaction and trust in recommendations. Yu [18] proposed a general framework based on graph regularization. The framework first extracts aspects from reviews, then models the user-item-aspect relationship as a tripartite graph, which can be used while performing Top-N recommendations, and provides various explanations. Sukrat and Papasratorn [19] developed a phrase-level sentiment analysis toolkit Sentiers1 that can extract user sentiment and product aspects from text reviews on a large scale. This toolkit can not only extract the “aspect-view-emotion” triples from the comments received by the item, for example, extract the “pixel-high-positive” and “carton-obvious-negative” three-tuples from the comments on the mobile phone tuple but also detect the emotion of the aspect word based on the context. For example, “pixel” and “high” indicate positive emotions, while “noise” and “high” indicate negative emotions. In addition, since the program constructs a dictionary of “aspect-view-emotion” triples, the toolkit can also detect the triples contained in the review text. Based on the Sentiers toolkit, Fedirko et al. [20] designed an explicit factor model and proposed to use the “aspect-view”the performance of the item in all aspects.

## 3. Intelligent Information Recommendation Algorithm Based on Deep Learning

**Figure 1:**
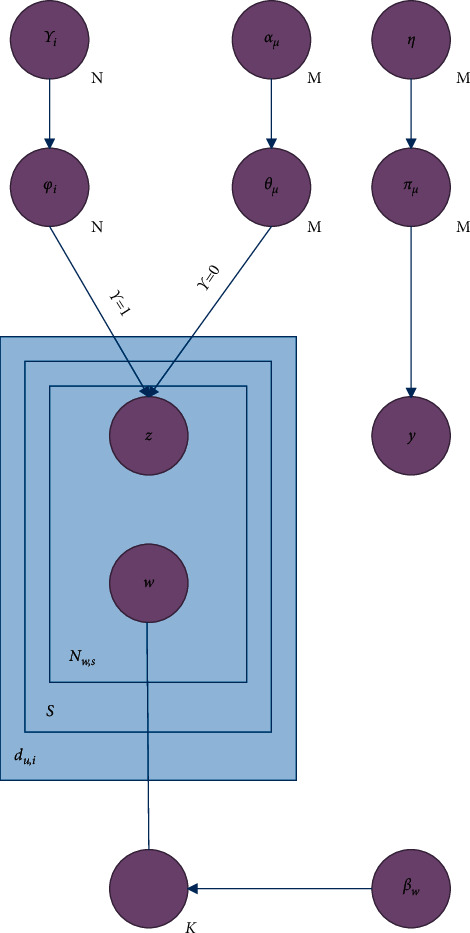
Text processor based on the topic model.

This paper proposes an improved topic model to solve the problem of feature extraction in the text of the recommendation system. The model is shown in Figure 1, where shaded circles represent observed variables and unshaded circles represent hidden variables. User *u* comments *d*_*u*,*i*_ ∈ *D* on item *i*. These generated sentences usually focus on a topic, either from the user's preference or from the characteristics of the item.

When the topic model is used as a text processor, very good performance can be obtained in the recommendation system. The main reason is that the topic model can simulate the process of manually writing comments, generate simulated comments based on user preferences and item characteristics, filter out some irrelevant content, and enhance the representation of users and items.

**Figure 2:**
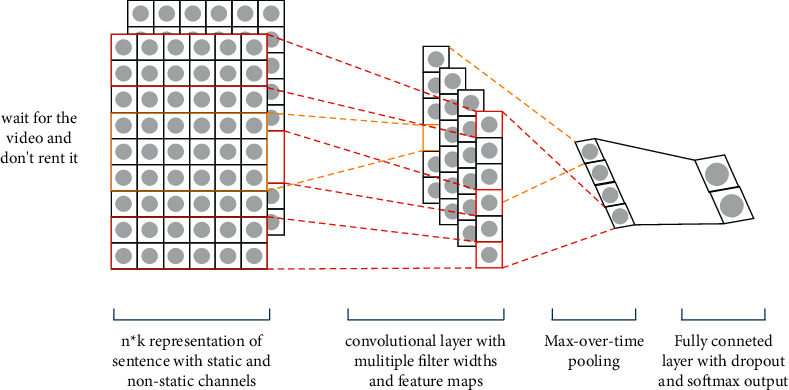
Text classification model based on convolutional neural network.

Convolutional neural network (CNN) is shown in Figure 2. Different from processing image data because text is a characteristic of one-dimensional data, CNN can only use one-dimensional convolution kernel *h* × *d* for convolution operation, that is, *d* is the dimension of a fixed word vector. Multiple convolution kernels with different values of *h* can obtain multiple feature maps, where *h* refers to convolution with *h* words as a window each time. Specifically, firstly, the convolution kernels with *h* being 2 and 3 are used for convolution to extract features. Then, the algorithm uses the maximum pooling operation on the obtained features. Finally, the algorithm combines the results of pooling in a splicing way as text features.

When a convolutional neural network is used as a text processor, the model has good performance in text classification and sentiment classification. The main reasons are as follows. The dimension of word vector can be kept within *d*, which avoids the problem of data sparseness and feature dimension disasters and also reduces the amount of model parameters. Convolutional neural networks can extract *h*-ary syntactic features. Convolutional neural networks can obtain more effective feature items in the feature by using the pooling layer and filter out certain noise to improve the accurate expression of features. The second layer is the convolutional layer, which contains *m* convolution kernels *K* ∈ *ℝ*^*h*×*d*^. The feature formula extracted by the *j*th neuron from the text is as follows:

(1) $$\begin{array}{c} z_{j}=\mathrm{ReLU}\left(w_{1:n}*K_{j}+b_{j}\right), \end{array}$$

where ∗ is the convolution operation, ReLU is the nonlinear activation function, and *b*_*j*_ is the bias. The text feature extracted by the *j*th element using the sliding window of *t* is *z*_1_, *z*_2_,…, *z*_*j*_^*n*−*t*+1^. The third layer is the maximum pooling layer, which can reduce dimensionality and capture the most important features. The formula is

(2) $$\begin{array}{c} o_{j}=\max\left(z_{1},z_{2},\ldots ,z_{j}^{n-t+1}\right). \end{array}$$

**Figure 3:**
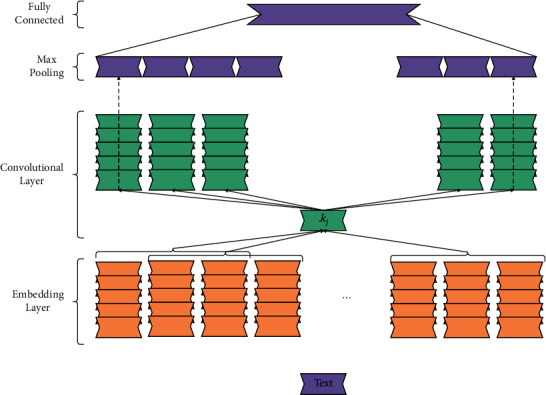
Text processor based on convolutional neural.

Finally, the features extracted by *m* neurons are pieced together to obtain text features *O*=(*o*_1_, *o*_2_,…, *o*_*m*_). The model is shown in Figure 3.

In the recommendation system, the text processor based on the convolutional neural network is adopted by many researchers. The experimental results prove that the text features it extracts can better express users and items, thereby improving the recommendation performance.

The research focus in natural language processing is how to extract rich features from sequence data. For example, in tasks such as translation systems, question answering systems, dialogue systems, and sentiment analysis, recurrent neural networks (RNN) are often used to encode or decode text. Among them, the most common recurrent neural networks for solving sequence problems are the long and short-term memory recurrent neural network. This section will take LSTM as an example to illustrate the process of recurrent neural network processing text.

**Figure 4:**
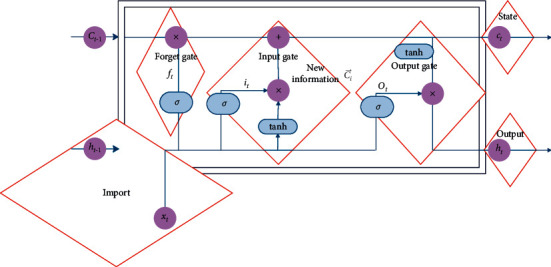
Unit model diagram of LSTM.

LSTM proposes to encode sentences according to the order of words. In addition to maintaining the sequence information of the text, it can also solve the problem of long-distance dependence. In addition, when LSTM considers the current word information, it also considers the historical code output by the last recurrent neural network unit. Specifically, LSTM has three gate mechanisms: input gate, forget gate, and output gate. The model is shown in Figure 4.

When coding word *x*_*t*_, the unit calculation process is shown in the following formula:

(3) $$\begin{array}{c} f_{t}=\sigma \left(W_{f}x_{t}+U_{t}\dot{h_{t-1}}+b_{f}\right),\\ i_{t}=\sigma \left(W_{i}x_{t}+U_{i}\dot{h_{t-1}}+b_{i}\right),\\ {ct}^{∼}=\tanh\left(W_{c}x_{t}+U_{c}\dot{h_{t-1}}+b_{c}\right),\\ c_{t}=f_{t}⊗c_{t-1}+i_{t}{⊗}_{c}^{∼}{\;}_{{\;}_{t}},\\ o_{t}=\sigma \left(W_{o}x_{t}+U_{o}\dot{h_{t-1}}+b_{o}\right),\\ h_{t}=o_{t}⊗\;\tanh\left(c_{t}\right). \end{array}$$

**Figure 5:**
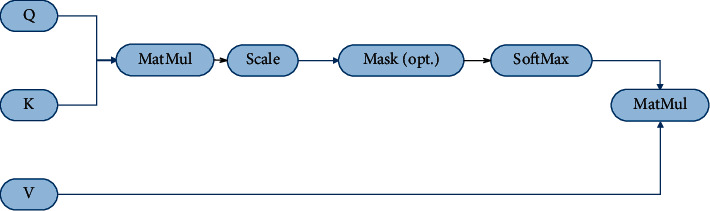
Model diagram of scaled dot-product attention.

Recurrent neural networks are commonly used to deal with sequence problems. However, due to the limitation of memory, the long-distance dependence of the cyclic neural network is still lost. Because the attention mechanism is not restricted by distance, it has become a popular technique for dealing with sequence problems. The text processor based on self-attention first converts the input matrix *X* into query matrix *Q*, key matrix K, and value matrix V, as shown in Figure 5. The calculation formula is as follows:

(4) $$\begin{array}{c} Q=XW^{Q},\\ K=XW^{K},\\ V=XW^{V}, \end{array}$$

where *W*_*q*_ ∈ *ℝ*^*d*×*d*_*k*_^, *W*_*k*_ ∈ *ℝ*^*d*×*d*_*k*_^, *W*_*v*_ ∈ *ℝ*^*d*×*d*_*v*_^, *Q* ∈ *ℝ*^*l*×*d*_*k*_^, *K* ∈ *ℝ*^*l*×*d*_*k*_^,  and *V* ∈ *ℝ*^*l*×*d*_*v*_^. Next, the algorithm calculates the dot product of query and key, divides the result by $\sqrt{d_{k}}$, and finally applies the softmax function to obtain the weight of value. The formula is as follows:

(5) $$\begin{array}{c} \mathrm{Attention}\left(Q,K,V\right)=\mathrm{softmax}\left(\frac{QK^{T}}{\sqrt{d_{k}}}\right)V. \end{array}$$

Because the target vector matrix Attention(*Q*; , *K*, *V*) calculated by this self-attention mechanism is obtained by point operation, that is, there is interaction between two words, therefore, using it as the coding matrix of the sentence itself, it can capture the dependency between any two words.

The self-attention text processor can extract the dependency relationship between words at any distance, and the execution efficiency is higher than that of the text processors of the convolutional neural network and the recurrent neural network.

**Figure 6:**
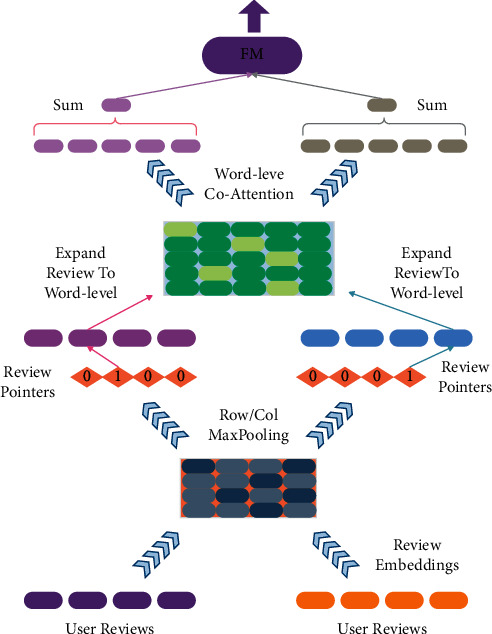
Co-attention model diagram.

As we all know, only part of the words or part of the comments in the comment-based recommendation is useful. There have been many works using the attention mechanism in conjunction with text processors of convolutional neural networks or cyclic neural networks to capture the usefulness of the word level or sentence level, but there is also a small amount of work that fully utilizes the attention mechanism to achieve this goal. The MPCN model is shown in Figure 6. MPCN uses the co-attention mechanism to deeply interact with user and item comments and captures the word-level and comment-level attention distribution. Specifically, the model first takes the encoded comment *x* ∈ *ℝ*^*l*×*d*^ as input and uses the co-attention network to first extract the comment-level co-attention. Among them, *l* is the length of the comment and *d* is the size of the word embedding. The formula is as follows:

(6) $$\begin{array}{c} s_{ij}=F{\left(x_{i}\right)}^{T}\text{MF}\left(x_{j}\right), \end{array}$$

where *i* and *j* represent the *i*th and *j*th comments, respectively, and *F*(.) is the feedforward neural network, *M* ∈ *ℝ*^*d*×*d*^,  and *S*=*s*_*ij*_ ∈ *ℝ*^*l*×*l*^. Next, the algorithm calculates comments *x*_*i*_ and *x*_*j*_ and considers the representations $\bar{x_{i}}$ and $\bar{x_{j}}$ of the comment-level co-attention; the formula is as follows:

(7) $$\begin{array}{c} \bar{x_{i}}={\left(\text{gumbel}\left(\max_{\text{col}}\left(S\right)\right)\right)}^{T}x_{i},\\ \bar{x_{j}}={\left(\text{gumbel}\left(\max_{\text{row }}\left(S\right)\right)\right)}^{T}x_{j}, \end{array}$$

where max_col_ and max_row _ are the maximum pooling operations performed on the columns and rows of the matrix S, respectively. The Gumbel function can obtain the one-hot encoded vector, that is, the useful comments are selected.

Then, the word-level co-attention is processed similarly. The algorithm takes ${\bar{x}}_{i}$ and $\bar{x_{j}}$ as input and outputs comments ${\bar{x}}_{i}$ and $\bar{x_{j}}$ that consider the word-level co-attention. The formula is as follows:

(8) $$\begin{array}{c} w_{ij}=F{\left(\bar{x_{i}}\right)}^{T}M_{w}F\left(\bar{x_{j}}\right),\\ \bar{x_{i}^{\prime }}={\left(\mathrm{softmax}\left({\mathrm{avg}}_{\mathrm{col}}\left(w\right)\right)\right)}^{T}\bar{x_{i}},\\ \bar{x_{j}^{\prime }}={\left(\mathrm{softmax}\left({\mathrm{avg}}_{\mathrm{row}}\left(w\right)\right)\right)}^{T}\bar{x_{j}}, \end{array}$$

where avg_col_ and avg_row_ are the average pooling operations performed on the columns and rows of the matrix *w*, respectively.

MPCN has greatly improved its performance on 24 publicly accessible datasets.

**Figure 7:**
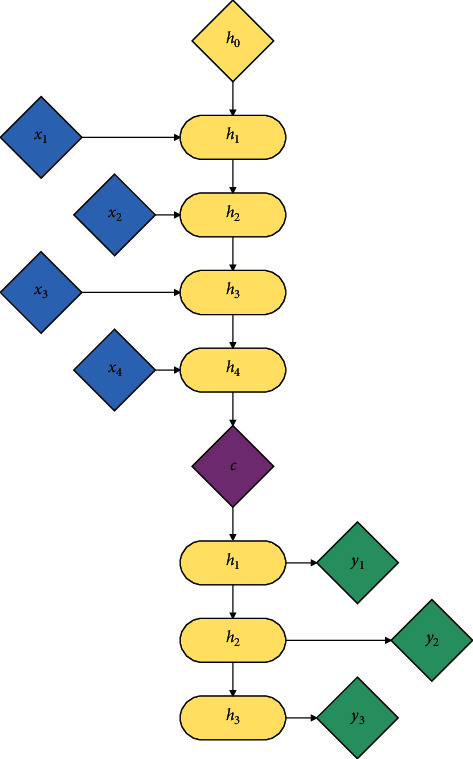
seq2seq model diagram.

In order to deal with the translation problem, an end-to-end sequence-to-sequence learning method is proposed. The model of this method is shown in Figure 7, which consists of two recurrent neural networks (RNN). One acts as an encoder to process the input sequence *x*_1_⟶*x*_2_⟶*x*_3_⟶*x*_4_, and the other acts as a decoder (sentence generator) to generate a sequence *y*_1_⟶*y*_2_⟶*y*_3_.

The specific steps are as follows:

(1) The algorithm uses the word embedding model to encode the input sequence and then enters the encoded words into the RNN one by one. The formula is as follows:
  (9) $$\begin{array}{c} h_{t}={\text{RNN}}_{\text{encoder}}\left(x_{t},h_{t-1}\right), \end{array}$$
where *x*_*t*_ is the input of the current unit, *h*_*t*−1_ is the output of the previous hidden unit, and *h*_*t*_ is the output of the current unit.
(2) The algorithm uses the output of the encoder as the first hidden state *h*_0_ of the decoder and uses the decoder to generate words one by one:
  (10) $$\begin{array}{c} h_{t}^{\prime }={\text{RNN}}_{\text{decoder}}\left({\hat{y}}_{t-1},h_{t-1}^{\prime }\right). \end{array}$$
(3) The algorithm uses the classifier to predict each word. The number of classifications is the size of the vocabulary. The formula is as follows:
  (11) $$\begin{array}{c} {\hat{s}}_{t}=\mathrm{softmax}\left(W{\hat{y}}_{t}+b\right). \end{array}$$

**Figure 8:**
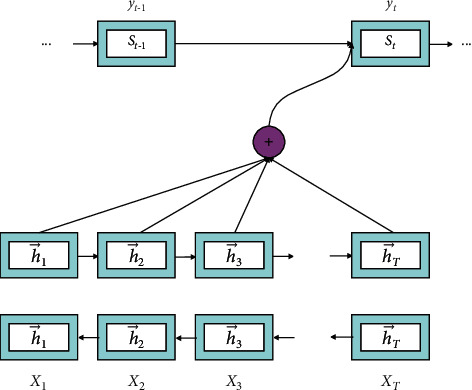
seq2seq model diagram based on attention mechanism.

The application of the seq2seq model has been widely used in other fields by translation systems, such as question answering systems, dialogue systems, and recommendation systems. Although LSTM and GRU are proposed to improve the dependence of words in sentences, the long-distance dependence is still not ideal. In order to solve this problem, the seq2seq joint attention mechanism is used to achieve better translation. The model is shown in Figure 8. The specific steps are as follows:

(1) The algorithm uses the word embedding model to encode the input sequence and then uses RNN to process the encoded words one by one. The formula is as follows:
  (12) $$\begin{array}{c} h_{t}={\text{RNN}}_{\text{encoder}}\left(x_{t},h_{t-1}\right). \end{array}$$
(2) The algorithm takes the output of the encoder as the first hidden state *h*_0_ of the decoder and uses the decoder to generate words one by one:
  (13) $$\begin{array}{c} s_{t}={\text{RNN}}_{\text{decoder}}\left({\hat{y}}_{t-1},s_{t-1}^{\prime }\right). \end{array}$$
(3) The algorithm uses the interaction between the hidden state of the decoder and the hidden state of the encoder to find the attention weight. The formula is as follows:
  (14) $$\begin{array}{c} \alpha_{ij}=\mathrm{softmax}\left(F\left(s_{i},h_{j}\right)\right). \end{array}$$
(4) The algorithm uses the attention weight to weight the hidden layer of the encoder as a context vector. The formula is as follows:
  (15) $$\begin{array}{c} c_{i}=\sum_{j=1}^{T}\alpha_{ij}h_{j}. \end{array}$$
(5) The algorithm concatenates the context vector and the hidden state of the decoder as the output of the predicted word and then inputs the result to the classifier to predict each word:
  (16) $$\begin{array}{c} P\left(y_{t}|y<t,x\right)=\mathrm{softmax}\left(W_{s}\tanh\left(W_{c}\left[c_{t};s_{t}\right]\right)\right). \end{array}$$

seq2seq combined with attention has better effect. The RNN unit of the seq2seq encoder and decoder can use a long short-term memory network (LSTM) or a gated recurrent unit (GRU), and the decoder is commonly used in recommendation systems as a generator of interpretable sentences. This study uses hierarchical GRU as a translator to generate natural language sentences, and the generated sentences can explain the recommended results well.

The formula for multihead attention is as follows:

(17) $$\begin{array}{c} {\text{head}}_{i}=\text{Attention}\left(QW_{i}^{Q},KW_{i}^{K},VW_{i}^{V}\right),\\ \text{MultiHea}\left(Q,K,V\right)=\text{Concat}\left({\text{head}}_{1},{\text{head}}_{2},{\text{head}}_{h}\right)W^{0}, \end{array}$$

where Concat (.) is the splicing operation. In a multihead decoder, K and V come from the output of the encoder and *Q* comes from the output of the upper layer of the decoder. Since attention is input in parallel, there is no position information between words, so this article proposes the position code of each word. The calculation formula is as follows:

(18) $$\begin{array}{c} PE_{pos,2i}=\frac{\sin\;\mathrm{pos}}{10000^{2i/d_{\text{model}}}},\\ PE_{pos,2i+1}=\frac{\cos\text{pos}}{10000^{2i/d_{\text{model}}}}, \end{array}$$

where pos is the position of the word, *i* is the dimension of the word, and *d*_model_ is the dimension of the position code.

The final output of the decoder can predict the probability of each word through a linear transformation function. The formula is as follows:

(19) $$\begin{array}{c} \text{FFN}\left(x\right)=\max\left(0,xW_{1}+b_{1}\right)W_{2}+b_{2},\\ {\hat{w}}_{x}=\mathrm{softmax}\left(\mathrm{FFN}\left(x\right)\right). \end{array}$$

The encoder and decoder models based on the self-attention mechanism further improve the effect of machine translation. The translation model is similar to seq2seq, in which the decoder can also be used as a sentence generator in the recommendation system to generate natural language sentences as the interpretation of the recommendation results.

## 4. Cross-Border e-Commerce Intelligent Information Recommendation System Based on Deep Learning

**Figure 9:**
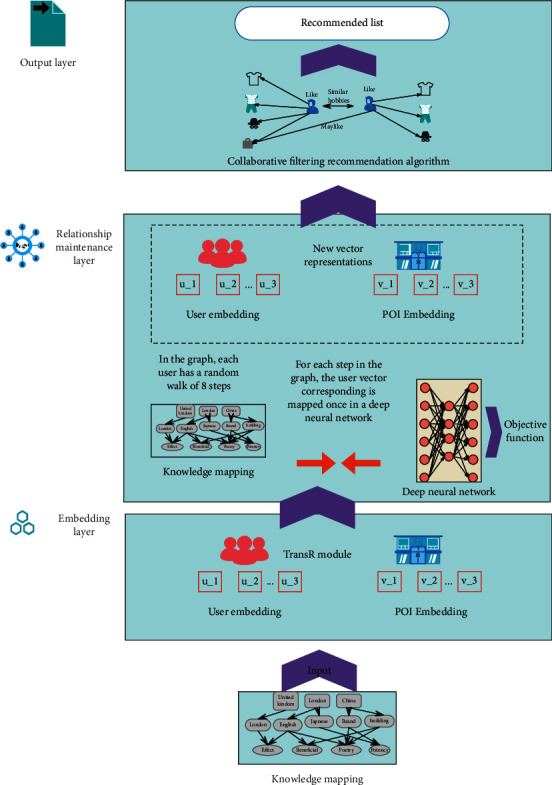
Cross-border e-commerce intelligent information recommendation system based on deep learning.

The cross-border e-commerce intelligent information recommendation system based on deep learning is shown in Figure 9. The model is divided into 3 layers. The first layer is the embedding layer, which initially expresses users and points of interest through the TransR model. The second layer is the relational reasoning layer, which forms a multilayer relational reasoning through random walks on the knowledge graph, calculates the information attenuation after each walk, and uses a deep neural network to perform a new vector mapping. Each mapping of a vector is a performance of relational information. Each mapping is concatenated to become the final vector representation of the user or point of interest, and the user set and the point of interest set are divided. The third layer is the output layer, which uses the vector representation of the new user's points of interest and recommends top-N points of interest to the user based on the collaborative filtering algorithm.

**Figure 10:**
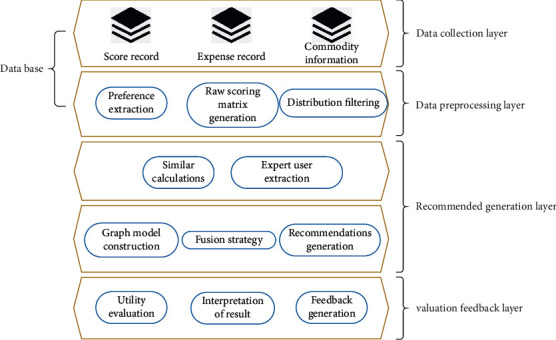
Recommendation system framework based on graph search strategy.

The new model constructed in this study links the status quo of various businesses in reality and changes the starting point of the previous recommendation system. We use the long tail theory to mine the mass commodities in the niche, recommend these products to users as suggestions, and combine the graph model to propose a niche commodity recommendation algorithm based on the graph search strategy. The specific framework is shown in Figure 10.

On the basis of the above analysis, the model proposed in this paper is verified. First of all, this article conducts simulation research on the effect of intelligent information processing and builds the system model of this article through simulation software to simulate the intelligent information processing of cross-border e-commerce. The results obtained are shown in Table 1.

From the above research, it can be seen that the method proposed in this paper can have a good effect. On this basis, the recommendation effect test is carried out, and the results shown in Table 2 are obtained.

Through the above experiments, it can be seen that the cross-border e-commerce intelligent information recommendation system based on deep learning proposed in this paper has a good recommendation effect and meets the recommendation needs of cross-border e-commerce.

## 5. Conclusion

With the continuous expansion of the scale of online platforms, online trading has become one of the indispensable activities on people's daily lives in the new era. This has also stimulated offline entities to a certain extent, forming an integrated online and offline sales strategy. With the increasing scale of online business platforms, recommendation systems have also risen, and they have become one of the backbone technologies in various fields today. It is also mainly from the perspective of ““people-oriented”“ to make the originally large and complex assembly line more intelligent, which greatly facilitates human beings. This paper applies deep learning to the cross-border e-commerce intelligent information recommendation system to overcome the geographical problems of cross-border e-commerce and improve the recommendation effect of cross-border e-commerce. The experiment shows that the cross-border e-commerce intelligent information recommendation system based on deep learning proposed in this paper has a good recommendation effect and meets the recommendation needs of cross-border e-commerce.

## Figures and Tables

**Table 1:** Intelligent information processing effect.

Number	Cross-border e-commerce information processing	Number	Cross-border e-commerce information processing
1	83.71	17	92.89
2	77.33	18	86.40
3	83.15	19	77.92
4	86.91	20	91.63
5	88.81	21	75.23
6	87.81	22	77.25
7	84.17	23	80.40
8	78.87	24	92.06
9	88.42	25	83.65
10	77.52	26	92.20
11	90.96	27	80.67
12	79.17	28	78.10
13	79.88	29	79.55
14	79.17	30	89.77
15	87.87	31	82.73
16	86.45	32	91.99

**Table 2:** Cross-border e-commerce intelligent information recommendation effect.

Number	Cross-border e-commerce information recommendation	Number	Cross-border e-commerce information recommendation
1	84.18	17	79.11
2	76.81	18	88.69
3	80.89	19	87.48
4	90.99	20	90.85
5	72.28	21	74.46
6	80.85	22	74.67
7	86.12	23	74.04
8	75.96	24	71.53
9	90.02	25	69.74
10	73.84	26	79.98
11	81.27	27	73.55
12	81.68	28	79.51
13	86.99	29	83.06
14	91.00	30	78.89
15	74.87	31	88.66
16	76.13	32	68.11

## Data Availability

The labeled dataset used to support the findings of this study are available from the corresponding author upon request.
